# Linking social support network deficits to late-life malnutrition: The hidden psychological pathway

**DOI:** 10.1016/j.jnha.2025.100686

**Published:** 2025-09-22

**Authors:** Miao Miao, Yu Zhou, Chen Qiu, Doris Sau Fung Yu

**Affiliations:** School of Nursing, Li Ka Shing Faculty of Medicine, The University of Hong Kong, Hong Kong, China

**Keywords:** Social support, Social network, Depressive symptoms, Malnutrition, Body mass index, Mediation analysis

## Abstract

**Objectives:**

Social and mental health significantly influence the nutritional status of older adults. While a relationship exists between social support network deficits and malnutrition, the underlying mechanisms remain unclear. This study aims to explore the associations and potential psychological pathways among social support network deficits, depressive symptoms, and malnutrition in older adults.

**Methods:**

A secondary analysis was conducted using data from the Jockey Club Pathway to Healthy Aging project, collected between May 2022 and June 2024. Structural equation modeling was employed to examine the relationships among social support network deficits, depressive symptoms, and malnutrition, as well as the mediating role of depressive symptoms in the overall participants and across body mass index subgroups.

**Results:**

The study included 5,286 older adults, with an undernourishment prevalence of 14.8%. Social support network deficits (β = −0.044, *p* = 0.004) and depressive symptoms (β = −0.150, *p* < 0.001) were significantly negatively associated with malnutrition scores, with depressive symptoms mediating 55.1% of the total effect (β = −0.098, *p* < 0.001). Depressive symptoms acted as a complete mediator in the normal weight group, while it served as a partial mediator in the overweight and obese groups.

**Conclusions:**

The risk of malnutrition in older adults is associated with social support network deficits and depressive symptoms. Preventive strategies should differ based on body mass index, focusing on social factors for overweight and obese individuals while addressing negative emotions in those with normal weight to encourage healthy dietary habits and lifestyles.

## 1 Introduction

Malnutrition is a significant public health issue among older adults, defined as a health condition of deficiencies, excesses, or imbalances in nutrient intake [1]. Globally, around 30% of older adults are at risk of malnutrition or already malnourished [2]. Substantial evidence indicates that malnutrition increases the risk of non-communicable diseases, accelerates physical and cognitive decline, contributes to emotional problems, and leads to premature mortality [3].

While extensive research has highlighted the role of lifestyle factors and macronutrients in late-life malnutrition [4], the importance of social determinants has been largely overlooked. Reviews have linked these determinants to food insecurity, inadequate social support, and low social integration, all of which contribute to unhealthy dietary choices, insufficient oral intake, reduced motivation for food-related activities [3,4]. More recently, the concept of social vulnerability has been introduced into the medical research to characterize the risk of adverse health outcomes influenced by broader social determinants operating across multiple levels—including individual, household, and community dimensions [5]. At the individual level, social support network deficits—reflecting a lack of diverse, stable, and responsive social ties—has emerged as a critical factor. It directly limits practical assistance with meal preparation, reduces opportunities for shared meals, and diminishes emotional encouragement necessary for maintaining adequate nutrition and eating routines in later life [[6], [7], [8]].

Whereas eating behaviors may be a crucial pathway to explain the connection of social support network deficits with non-disease-related malnutrition among older adults, the negative emotional response such as depressive symptoms associated with economic deprivation [9], reduced social participation [10], and lack of social support [11] may suggest a potential psychological pathway. Notably, older adults with different body mass indices (BMI) may exhibit varying tendencies and dietary behaviors in response to negative emotions [12]. Evidence indicates that obese older individuals are more likely to engage in emotional eating compared to normal-weight counterparts [12]. Furthermore, a connection between depressive symptoms and overeating [13], as well as a sedentary lifestyle among older adults [14], has been established. This raise concerns that, malnutrition, typically characterized by reduced food intake, unintentional weight loss, and low BMI, may also manifest as over-nutritional intake and resultant obesity. Therefore, we hypothesized that social support network deficits are associated with the nutritional status of older adults through the mediating role of depressive symptoms (Fig. S1). The associations of social support network deficits and depressive symptoms with malnutrition would differ across different BMI subgroups.

## 2 Methods

### 2.1 Data

This secondary analysis was part of the Jockey Club Pathway to Healthy Aging (PathHA), a territory-wide project in Hong Kong that employed the World Health Organization Integrated Care for Older Persons (WHO-ICOPE) model to promote healthy aging. Eligible participants were aged 60 or older, able to communicate with research personnel, and provided informed consent. From May 2022 to June 2024, a total of 5,286 older adults were recruited through convenience sampling for this analysis.

### 2.2 Social support network deficits

The 3-item Tilburg frailty indicator (TFI) social sub-scale was used to measure social support network deficits, including living alone, inadequate social companionship, and insufficient social support. All items use a dichotomous scale, where higher scores indicate greater severity [15]. We extracted corresponding items from the PathHA database to assess these dimensions, re-coding scores to align with the TFI sub-scale (See Table S1). The Kuder-Richardson Formula 20 (KR-20) of this sub-scale was 0.32 in the current sample. Sensitivity analysis revealed low inter-item correlations (ranging 0.101–0.191), and the KR-20 remained unchanged after removing insufficient social support (See Table S1.1) indicating that the modest reliability was not due to any specific item. These findings align with previous studies reporting KR-20 values between 0.22–0.49 for similar measures [16]. Though the brevity of the scale may account for this value of internal consistency, its strong test-retest reliability (r = 0.76) supports psychometric adequacy [15].

### 2.3 Depressive symptoms

Depressive symptoms were measured using the 15-item Geriatric Depression Scale (GDS-15). Higher scores indicate greater depressive symptom severity, with cut-off scores as follows: 0–4 for normal mood, 5–8 for mild depressive symptoms, and 9 or above for moderate to severe depressive symptoms. GDS-15 was found to have good specificity and sensitivity to identify late-life depressive symptoms [17]. The Cronbach’s alpha was 0.81 in the current sample.

### 2.4 Malnutrition

The Mini-Nutritional Assessment-Short Form (MNA-SF) was used to measure the nutritional status. Its sensitivity, specificity, and diagnostic accuracy are comparable to the full MNA [18]. The total score ranges from 0 to 14, with higher scores indicating better nutritional status. For this study, scores were further categorized into two groups: normal (12–14 points) and undernourished (0–11 points). The Cronbach’s alpha for this study was 0.63.

### 2.5 BMI

Following the recommendations for Asia-Pacific population by WHO [19], participants were classified into four BMI groups: underweight (<18.5 kg/m^2^), normal weight (18.5–22.9 kg/m^2^), overweight (23.0–24.9 kg/m^2^), and obese (≥25 kg/m^2^).

### 2.6 Covariates

The demographic characteristics, including age, gender, education level, and marital status, were extracted as covariates from the PathHA dataset.

### 2.7 Statistical analysis

Descriptive statistics, including percentages, means, and standard deviations (SD), summarized demographic data. The Chi-square (χ^2^) test and independent samples t-test compared differences in demographic factors, social support network deficits, and depressive symptoms based on nutritional status, using IBM-SPSS® version 26, with a significance level set at p < 0.05.

Structural equation modeling (SEM) tested the hypothesized model in Mplus Editor version 1.6. Model robustness was evaluated using fit indices: a non-significant χ^2^, a Root Mean Square Error of Approximation (RMSEA) < 0.08, Tucker-Lewis Index (TLI) and Comparative Fit Index (CFI) values ≥ 0.90, and a Standardized Root Mean Square Residual (SRMSR) ≤ 0.10. Multi-group SEM was employed to test structural invariance by comparing the chi-square (Δχ^2^) and degrees of freedom (Δ df) between constrained and unconstrained models. Furthermore, path coefficients across BMI subgroups were compared using Wald χ^2^ tests.

## 3 Results

### 3.1 Sample characteristics

The mean age of the 5,286 participants was 73.4 (SD = 7.3), with females comprising 77.3% of the sample. Undernourished participants (n = 783) had lower education levels, were more likely to be unmarried, exhibited higher social support network deficits and more severe depressive symptoms compared to those with normal nutrition (n = 4,503) (See Table 1).

**Table 1:** Demographic and health-related characteristics of the participants stratified by nutritional status.

Variables	Total	Normal	Undernourished	χ²/t
	n = 5,286	n = 4,503 (85.2%)	n = 783 (14.8%)	
Age (Mean ± SD)	73.4 ± 7.32	73.34 ± 7.23	73.75 ± 7.75	−1.354^a^
Sex, n (%)				
Male	1,200 (22.7%)	1039 (86.6%)	161(13.4%)	2.398^b^
Female	4,086 (77.3%)	3464 (84.8%)	622 (15.2%)	
Education level, n (%)				
Never educated or kindergarten	582 (11.0%)	518 (89.0%)	64 (11.0%)	11.043^b*^
Primary	2097 (39.7%)	1795 (85.6%)	302 (14.4%)	
Junior or high School	2219 (42.0%)	1871 (84.3%)	348 (15.7%)	
Tertiary education	388 (7.3%)	319 (82.2%)	69 (17.8%)	
Marital status, n (%)				
With spouse	2817 (53.3%)	2434 (86.4%)	383 (13.6%)	7.075^b*^
Without spouse	2469 (46.7%)	2069 (83.8%)	400 (16.2%)	
TFI-social (Mean ± SD)	0.7 ± 0.8	0.64 ± 0.79	0.84 ± 0.89	−5.865^a***^
GDS-15 (Mean ± SD)	3.0 ± 3.1	2.81 ± 2.93	3.97 ± 3.69	−8.379^a***^
Normal, n (%)	3978 (75.3%)	3479 (87.5%)	499 (12.5%)	89.618^b***^
Mild depressive symptoms, n (%)	935 (17.7%)	756 (80.9%)	179 (19.1%)	
Moderate to severe depressive symptoms, n (%)	373 (7.1%)	268 (71.8%)	105 (25.2%)	

Notes: SD: standard deviation. TFI: Tilburg Frailty Indicator. GDS: Geriatric Depression Scale. a Comparison between the groups through independent samples t-test, reporting the t-value. b Comparison between the groups through the χ^2^ test reported Pearson χ^2^ values. * p < 0.05, ** p < 0.001, *** p < 0.001.

### 3.2 Mediating effect of depressive symptoms on social support network deficits and malnutrition

**Fig. 1:**
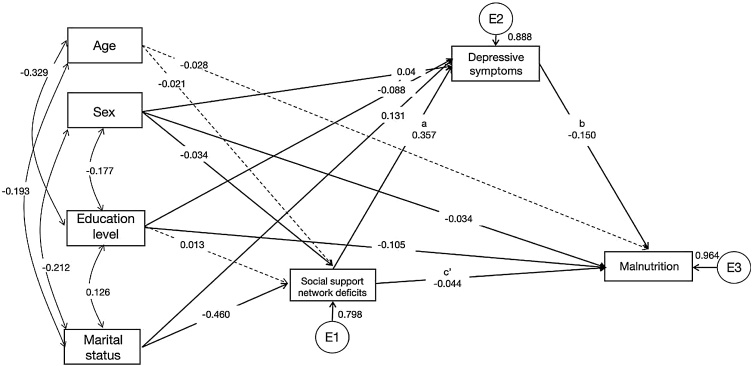
Path diagram for the mediating effect of depressive symptoms in the association between social support network deficits and malnutrition for the overall sample. Continuous line: significant parameter. Dash line: non-significant parameter. Single arrow path: standardized regression coefficient. The double-headed arrow path: standardized correlation coefficient. E1–E3: error terms.

Fig. 1 and Table S2 show the path diagram and the standardized estimation of the relationship between social support network deficits, depressive symptoms, and malnutrition score in the overall sample. The goodness-of-fit of the model was acceptable (See Table S3). After adjusting for all potential covariates, a significant partial mediating effect of depressive symptoms was observed in the relationship between social support network deficits and malnutrition (β = −0.054, p < 0.001).

The multi-group SEM analysis of BMI categories showed acceptable model fit. No significant differences were found between constrained and unconstrained models (Δχ^2^ = 11.907, Δ df = 9, *p* = 0.219), supporting structural invariance (Table S3).

After adjusting for covariates, the total effect of social support network deficits on malnutrition was not significant in the underweight group (n = 252), indicating that the mediation hypothesis does not apply to this subgroup. In contrast, for the normal weight group (n = 1,964), depressive symptoms served as a complete mediator. In the overweight and obese groups, depressive symptoms partially mediated the relationship between social support network deficits and malnutrition (See Table S4). Table 2 indicates that the indirect effect in the normal weight group was greater than in the obese group (Z = 4.455, *p* = 0.035), suggesting that the mediating role of depressive symptoms weakened in obese individuals.

**Table 2:** Comparison of standardized path effects across Asian-Pacific BMI subgroups.

	Total effect (c)		Indirect effect (a*b)		Direct effect (c’)	
	Z	*p*^	Z	*p*^	Z	*p*^
G2 vs G3	1.783	0.182	1.204	0.272	2.906	0.088
G2 vs G4	0.828	0.363	4.455	0.035	2.773	0.096
G3 vs G4	0.263	0.608	0.788	0.375	0.042	0.838

Notes: Z: Z-score of the Wald χ^2^ test, representing the ratio of differences in path coefficients to their standard errors. ^ The p-value is derived from the Wald χ^2^ test. G2: Normal weight group. G3: Overweight group. G4: Obese group.

## 4 Discussion

Our findings not only confirm the association of social support network deficits with the nutritional status of older adults and the mediating role of depressive symptoms but also highlight the varying expressions of social and psychological factors across different BMI subgroups. Therefore, addressing malnutrition in older adults requires moving beyond age-related physiological decline and access to food or social resources, and adopting a more holistic approach that considers the link between social deficits and both psychological well-being and dietary behaviors.

This study found that social support network deficits are associated with an increased risk of malnutrition among community-dwelling older adults, which is consistent with previous research findings [20]. Prior studies have identified living status, loss of social connections, and insufficient social support as risk factors for malnutrition [8,21,22], respectively. Indeed, these social factors are often interconnected, complicating the identification of specific social mechanisms leading to malnutrition. For example, older adults living with their families may still eat alone due to poor family relationships and different life patterns [23]. Similarly, healthy older adults living alone may face a lower risk of malnutrition than those unable to live independently and lacking social support [24]. Therefore, using a multidimensional concept such as social support network deficits allow for a more accurate capture of social complexity in high-vulnerability populations.

Although social vulnerability and social support network deficits are related, they reflect distinct dimensions of social deficits. Social vulnerability emphasizes systemic deficiencies in resources and capacities that impair an individual’s ability to cope with health crises [25]. In contrast, social support network deficits operate at the individual level, characterized by insufficient interpersonal connections and a lack of social support, which otherwise serve as buffers against health deterioration and psychological distress. Importantly, exposure to a highly vulnerable social environment increases an individual’s risk of developing social support network deficits [26]. These insights highlight the need for integrated interventions that address both community-level systemic weaknesses and individual-level social deficits, tailored to specific contextual vulnerabilities.

However, the potential for reverse causality must be considered. It is plausible that malnutrition itself, leading to physical frailty, sarcopenia [27], and depression [28], may reduce an individual’s motivation and capacity to engage socially, thereby exacerbating social support network deficits. This bidirectional relationship suggests that social deficits and malnutrition may form a vicious cycle in later life. Therefore, future longitudinal studies are crucial to disentangle the temporal sequence and confirm the directionality of this association.

Another finding of this study is that depressive symptoms mediate the association between social support network deficits and malnutrition among community-dwelling older adults. Social support network deficits significantly are associated with depressive symptoms [29]. Furthermore, depressive symptoms are associated with malnutrition [30], often co-occurring with eating disorders [31]. On the one hand, disruption of the reward pathway manifests itself in some individuals as anorexia [32], leading to a decrease in the quality and quantity of food intake. On the other hand, reduced stress activity in the hypothalamic-pituitary-adrenal axis might contribute to abnormal increases in appetite [33]. As a result, individuals may engage in emotional eating, raising the risk of excessive energy intake and nutrient deficiencies [31].

Interestingly, our findings indicate that social support network deficits are not significantly associated with malnutrition in the low BMI group. Unintentional weight loss in older adults is frequently related to various medical conditions, including malignant tumors, non-malignant gastrointestinal disorders, acute illnesses, oral health issues, and mental health conditions such as anorexia of aging [34]. These factors can elevate energy expenditure or disrupt nutritional intake, contributing to low BMI and malnutrition.

In the normal weight group, social support network deficits increase the risk of malnutrition entirely through depressive symptoms. This finding highlights the importance of mental health in maintaining nutritional status among individuals with normal weight, suggesting that nutritional interventions for this group should prioritize the assessment and improvement of mental health. In contrast, for the overweight and obese groups, social factors exert a more pronounced direct association with malnutrition. This suggests that their nutritional status is closely tied to social relationships, which may shape dietary choices and lifestyle [35]. Consequently, nutritional intervention for these populations must consider the role of social contextual factors to promote healthier eating habits and lifestyle changes.

## 5 Limitation

While our study benefited from a large sample size, several limitations warrant consideration. Its cross-sectional design precludes causal inference. Measurement issues may also arise, as the MNA-SF could underestimate malnutrition in overweight/obese older adults. Furthermore, our assessment of social support network deficits was insufficiently comprehensive, as it failed to capture the specific types or cumulative burden of support impairments, potentially limiting the findings’ scope. The generalizability of our results is also constrained by the urban Chinese sample, as the social support network deficits-malnutrition pathway may differ in regions with stronger social support systems or greater resource limitations, where cultural and environmental factors (e.g., familial structures, food access, community development) could significantly influence outcomes. Future studies should employ longitudinal designs, more precise nutritional tools, and multidimensional social vulnerability assessments to facilitate cross-cultural comparisons.

## 6 Conclusion

This study emphasizes the importance of addressing both social support network deficits and emotional well-being in strategies aimed at improving nutritional health in later life. Prevention and management of malnutrition should be tailored to the varying BMI categories among older adults. Health professionals can create individualized interventions that consider the specific social, psychological, and nutritional needs, promoting early intervention and reducing the risk of malnutrition.

## CRediT authorship contribution statement

Doris Sau Fung YU: Conceptualization, Funding acquisition, Supervision, Writing - Review & Editing; Miao MIAO: Methodology, Formal analysis, Writing - Original Draft; Yu ZHOU: Writing - Review & Editing; Chen QIU: Methodology. All authors read and approved the final manuscript. All authors reviewed and approved the final manuscript.

## Ethical statement

This study was approved by the Institutional Review Board of the University of Hong Kong/Hospital Authority Hong Kong West Cluster (Ethics No. UW 22-277). Informed consent was obtained from all subjects involved in the study. Written informed consent has been obtained from the patients to publish this paper.

## Funding statement

This research was funded by the Hong Kong Jockey Club Charities Trust. (Project title: Jockey Club Pathway to Healthy Aging, Project Number: S/N#2021/0328).

## Data availability

The data presented in this study are available on request from the corresponding author due to privacy and ethical reasons.

## Declaration of competing interest

The authors declare no competing interest.
