# Supplementary material for: Linking social support network deficits to late-life malnutrition: The hidden psychological pathway
Source: J Nutr Health Aging. 2025 Sep 22;29(10):100686. doi: 10.1016/j.jnha.2025.100686 (PMC12494835; doi:10.1016/j.jnha.2025.100686)
Supplement: Supplementary file 1 [file mmc1.docx]

**Appendix A: Supplementary materials**


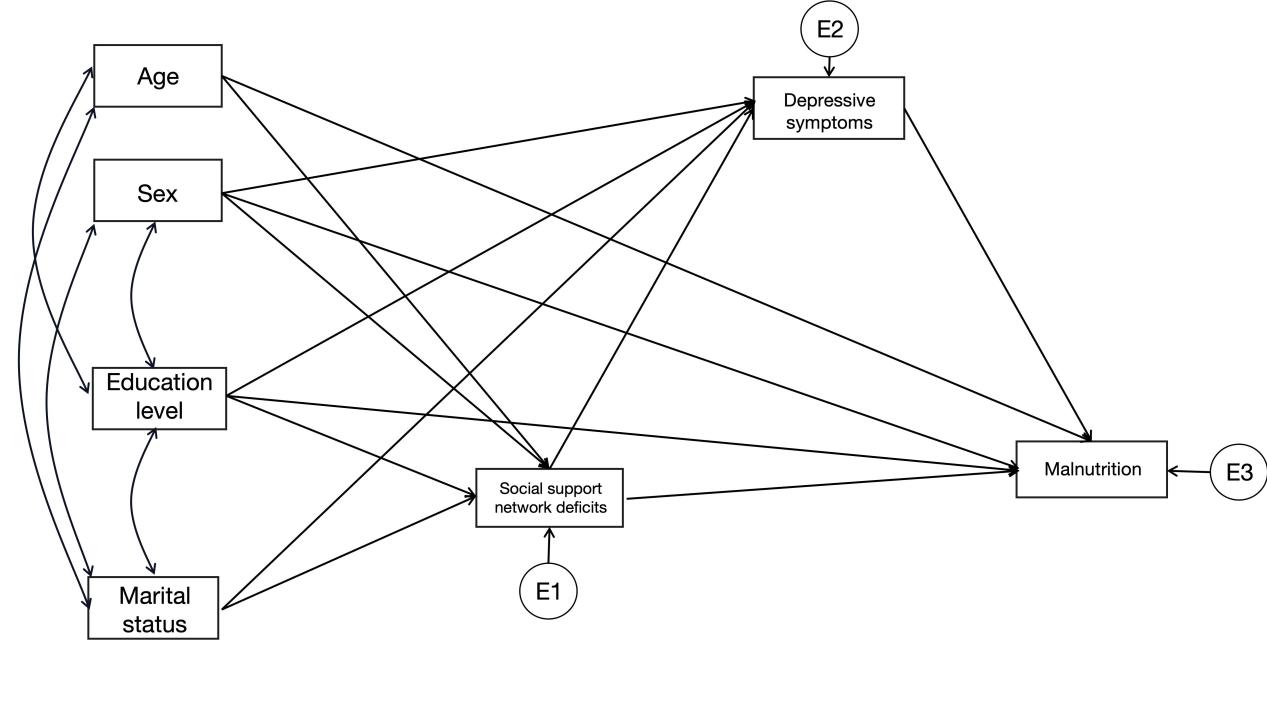


**Figure S1.** **Hypothesized tested model.** Age, sex, educational level, and marital status are covariates. The straight lines represent causal relationships, while the arrows indicate the direction of influence. Curved double arrows signify the existence of correlations between variables. E1–E3: error terms.

**Table S1.** TFI social sub-scale and extracted items

| **TFI social sub-scale** | **Item (Scale)** | **Scoring** |
| --- | --- | --- |
| Living alone | Do you live alone? (Demographic profile) | No = 0, Yes = 1 |
| Social companionship | How often do you feel that you lack companionship? (University of California, Los Angeles Loneliness Scale) | Never = 0, Always and Sometimes = 1 |
| Social support | When you need help, can you count on someone willing and able to meet your needs? (Edmonton Frail Scale) | Always = 0, Sometimes and Never = 1 |

**Table S1.1** TFI social subscale inter-item correlation and sensitivity analysis

|  | **Living alone** | **Inadequate social companionship** | **Insufficient social support** | **KR-20 if item deleted** |
| --- | --- | --- | --- | --- |
| Living alone | 1.000 | - | - | 0.183 |
| Inadequate social companionship | 0.191 | 1.000 | - | 0.174 |
| Insufficient social support | 0.101 | 0.104 | 1.000 | 0.319 |

**Table S2.** The mediating effect of depressive symptoms on social support network deficits and malnutrition for the overall sample.

| **Effect** | **β** | **SE** | **Est./S.E.** | ***p*** | **BootLL CI** | **BootUL CI** |
| --- | --- | --- | --- | --- | --- | --- |
| Total effect (c) | -0.098 | 0.015 | -6.405 | < 0.001 | -0.129 | -0.069 |
| Indirect effect (a*b) | -0.054 | 0.006 | -8.285 | < 0.001 | -0.067 | -0.042 |
| Direct effect (c’) | -0.044 | 0.015 | -2.916 | 0.004 | -0.076 | -0.015 |
| Mediation proportion (a*b/c) | 0.551 |  |  |  |  |  |

Note: The model was adjusted for age, sex, education level, and marital status. β, standardized coefficients. SE, Standard error. Est./S.E., the ratio of estimate and standard error. BootLL, Bootstrapping lower limit. BootUL, Bootstrapping upper limit. CI, confidence interval.

**Table S3.** Measurement model fit results

| **Model Fit** | **Model (Overall)** | **Multi-group Model (Unconstrained)** | **Multi-group Model (Constrained)** |
| --- | --- | --- | --- |
| χ2(*p*) | 4.310 (0.116) | 8.662 (0.372) | 20.569 (0.246) |
| RMSEA | 0.015 | 0.008 | 0.013 |
| CFI | 0.999 | 0.999 | 0.995 |
| TLI | 0.991 | 0.995 | 0.988 |
| SRMR | 0.005 | 0.009 | 0.015 |

**Table S4.** The mediating effect of depressive symptoms on social support network deficits and malnutrition grouped by Asia-Pacific BMI.

| **Subgroup** | **Effect** | **β** | **SE** | **Est./S.E.** | **p** | **BootLLCI** | **BootUL**  **CI** |
| --- | --- | --- | --- | --- | --- | --- | --- |
| G1 | Total  effect (c) | -0.078 | 0.063 | -1.244 | 0.213 | -0.201 | 0.045 |
| G2 | Total  effect (c) | -0.079 | 0.023 | -3.493 | < 0.001 | -0.123 | -0.035 |
|  | Indirect effect (a*b) | -0.062 | 0.010 | -6.557 | < 0.001 | -0.081 | -0.044 |
|  | Direct  effect (c’) | -0.017 | 0.024 | -0.702 | 0.483 | -0.063 | 0.030 |
|  | Mediation proportion (a*b/c) | 0.785 |  |  |  |  |  |
| G3 | Total  effect (c) | -0.127 | -0.182 | -4.550 | < 0.001 | -0.182 | -0.072 |
|  | Indirect effect (a*b) | -0.047 | 0.010 | -4.878 | < 0.001 | -0.066 | -0.028 |
|  | Direct  effect (c’) | -0.080 | 0.029 | -2.795 | 0.005 | -0.136 | -0.024 |
|  | Mediation proportion (a*b/c) | 0.370 |  |  |  |  |  |
| G4 | Total  effect (c) | -0.109 | 0.023 | -4.695 | < 0.001 | -0.154 | -0.063 |
|  | Indirect effect (a*b) | -0.036 | 0.008 | -4.628 | < 0.001 | -0.052 | -0.021 |
|  | Direct  effect (c’) | -0.072 | 0.024 | -3.059 | 0.002 | -0.119 | -0.026 |
|  | Mediation proportion (a*b/c) | 0.330 |  |  |  |  |  |

The model was adjusted by age, sex, education levels, and marital status. β, standardized coefficients, Standard error; Est./S.E., the ratio of estimate and standard error; BootLL, Bootstrapping lower limit; BootUL, Bootstrapping upper limit; CI, confidence interval. G1: Underweight group. G2: Normal weight group. G3: Overweight group. G4: Obese group.
